# Plant polyphenols, terpenes, and terpenoids in oral health

**DOI:** 10.1515/med-2025-1183

**Published:** 2025-04-15

**Authors:** Vida Arzani, Mohsen Soleimani, Tilman Fritsch, Ursula M. Jacob, Vittorio Calabrese, Ahmad Arzani

**Affiliations:** School of Dentistry, Iran University of Medical Sciences, Tehran, Iran; Department of Pharmacology, School of Medicine, AJA University of Medical Sciences, Tehran, Iran; NAM Institute, Rochusgasse 13, 5020, Salzburg, Austria; Healthcare AG, Zurich, CH, Switzerland; Department of Biomedical and Biotechnological Sciences, University of Catania, 95124, Catania, Italy; Department of Agronomy and Plant Breeding, College of Agriculture, Isfahan University of Technology, Isfahan, 8415683111, Iran

**Keywords:** bioactive compounds, antimicrobial, dental caries, gingivitis, periodontitis

## Abstract

**Introduction:**

Herbal products are increasingly being used for their potential to promote oral health. Phytochemicals can act as scavengers of reactive or toxic chemicals, selective growth factors for beneficial oral bacteria, fermentation substrates for beneficial oral bacteria, and selective inhibitors of harmful oral microflora. Plant-derived secondary metabolites encompass a variety of natural products, including alkaloids, polyphenols (including flavonoids and tannins), terpenes, terpenoids, steroids, saponins, quinones, coumarins, glycosides, and carotenoids.

**Objectives:**

This review explores medicinal plant-based products for preventing and treating oral diseases, offering updated insights into the scientific basis for using herbs as active ingredients in oral health care. Hence, we focused on: (1) phenolic compounds, the most abundant and common phytochemicals and (2) terpenes and terpenoids, the most diverse and widely distributed group in the plant kingdom.

**Methods:**

Several databases were queried to acquire peer-reviewed studies focusing on the major phytochemical compositions – phenolic compounds, terpenes, and terpenoids – and their oral health benefits.

**Results:**

The review lists numerous medicinal herbs in various forms and applications for treating dental caries, gingivitis, and periodontitis.

**Conclusion:**

This review explores the evidence supporting the oral health-promoting effects of specific constituents in herbs with the potential for incorporation into pharmaceutical formulations.

## Graphical abstract

**Figure:**
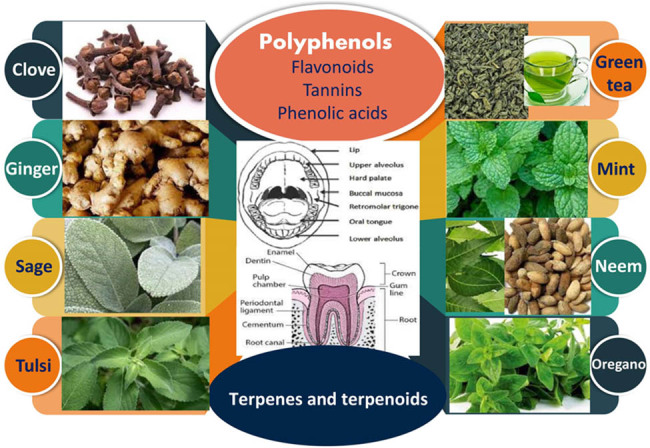

## 1 Introduction

Oral disorders, including dental caries, gingivitis, periodontitis, and oral infections, are highly prevalent diseases that affect more than 3.5 billion people worldwide [1]. These conditions not only impose significant health and economic burdens but also lead to a decline in patients’ quality of life. Dental caries, tooth loss, periodontal disease, and oral cancers are among the most common and significant oral diseases worldwide. Furthermore, due to their progressive and chronic nature, these oral diseases can become lifelong conditions. It should also be noted that periodontal tissues and teeth play a vital role in the overall functioning of the human body [2]. The oral cavity is continuously exposed to the external environment through contact with water, food, and air. This environment harbors a diverse and vast array of microorganisms, including bacteria, archaea, mycoplasmas, fungi, protozoa, and viruses. Disruption of the balance and homeostasis between pathogenic and beneficial microorganisms is believed to be one of the leading causes of oral diseases [3].

Recently, there has been growing global interest in the potential of plant-derived products as therapeutic agents for the prevention and/or treatment of oral diseases. Natural antioxidants from plant sources are gaining particular attention as substitutes for synthetic antioxidants because they offer various health benefits, such as prevention, vitality, and treatment [4]. Bacterial antibiotic resistance remains a significant health challenge both in the United States [5] and globally [6]. The literature in this field is extensive. However, a recent systematic review conducted by Brooks et al. [7] examined the occurrence and prevalence of antibiotic-resistant genes in the oral cavity. Antibiotic resistance genes are widespread among bacteria found in the oral cavity, with tetracycline resistance genes (such as *tet(M)*) being the most common. Phytodentistry, on the other hand, involves the use of botanical drugs to prevent or treat oral diseases. Further research on novel antimicrobial agents, including antibiofilm agents for biofilm-related infections, is crucial for developing effective strategies against antibiotic-resistant bacteria, and the chemically complex nature of plant-derived antimicrobial agents offers a promising alternative [4]. Botanical antibiotics primarily function by disrupting the plasma and intracellular membranes and cell walls, causing the release of the cytoplasmic contents of microorganisms, which ultimately results in their death [8]. Phytochemical constituents also exhibit antibacterial activity by potentially inhibiting bacterial enzymes, including those involved in sortase, DNA replication, toxins, and cell lysis [4,9,10]. Another significant contribution of phytochemicals to oral health is their presence in balanced diets and food supplies. This perspective aligns with firmly established evidence, consistently suggesting that societies adhering to diets rich in phytochemicals tend to have notably improved oral health [11].

Secondary metabolites encompass a huge variety of bioactive compounds known as phytochemicals. These chemical compounds accumulate in cells to defend against a vast array of biotic (pathogens and insects) and abiotic (such as drought, frost, salt, and heat) stressors in plants [12]. They are produced from diverse primary metabolites directly or indirectly through biosynthetic intermediates. Phenolic compounds, including polyphenols and phenolic acids, constitute one of the largest and most widely distributed phytochemical groups in vascular plants [4]. However, another noteworthy category of phytochemicals comprises terpenes and terpenoids, representing the most diverse and broadly distributed group across the plant kingdom [13]. There are substantial bodies of literature focusing on phenolic compounds in edible plant parts, mainly because of the interest in the health benefits of polyphenol consumption [4]. Phenolic compounds benefit health due to their chemopreventive activities, such as anticarcinogenic, antimutagenic, and antioxidative potential [4]. Recently, dietary polyphenols have received much attention due to their therapeutic and preventive potential for many chronic diseases, including diabetes, cancer, chronic inflammation, cardiovascular disease, and neurodegenerative diseases [14]. Dietary polyphenols are the most abundant phytochemical compounds in plants and plant-based foods and beverages, including vegetables (herbs), fruits, cereals, nuts, coffee, and tea. Polyphenols in one’s diet are also present in bee-derived products such as honey, propolis, beeswax, and bee bread. However, the amount and quality of these polyphenols predominantly rely on the pollen and nectar plants from which bees collect their resources.

In addition, plant-derived versatile products are in high demand in the cosmetics, food, and pharmaceutical industries due to their safety, lower risk, relatively low extraction cost, ease of product storage, and ease of scale-up. The emergence of plant molecular farming has revitalized global interest in producing valuable natural bioactive molecules and pharmaceutical proteins in biofactory plants used as bioreactors [15].

General health and oral health are intimately linked to each other [16]. The oral cavity acts as a window to many body systems and contains a unique oral microbiome comprising over 700 species of bacteria [17]. It is widely recognized that various oral diseases and conditions, such as dental caries and periodontal diseases, can be associated with numerous other health conditions, including cardiovascular diseases, diabetes, endocarditis, dementia, and cancer [1].

For example, maintaining good oral hygiene can significantly reduce the risk of pneumonia, decreasing the colonization of respiratory pathogens in the oral cavity and subsequent aspiration into the lower airway. Additionally, there is growing evidence of the health and therapeutic benefits of phytochemicals derived from herbs, specifically oral tissues [4]. These phytochemicals offer numerous advantages, serving as potential sources of nonenzymatic antioxidants such as polyphenols and vitamins (A, E, and C). These antioxidants neutralize the free radicals produced in oral mucosal lesions, thereby preventing mucosal damage. Moreover, phytochemicals have been found to modulate cellular signaling pathways, activating cellular defense mechanisms that protect normal cells against reactive oxygen species (ROS) or reactive nitrogen species. They can also induce apoptotic death in cancerous cells, thus contributing to chemoprevention by preventing, reversing, or delaying carcinogenesis [18].

A wide variety of diseases and disorders impair the hard and soft tissues of the mouth and may give rise to a range of congenital anomalies, craniofacial disorders, injuries, and various infections. However, dental caries and periodontal diseases are the key clinical conditions that prioritize global public health [1].

Dental caries occurs when acidic byproducts of bacteria ferment free sugars, causing localized destruction of the hard tissues (enamel and dentine) of the teeth [19]. As caries become more aggressive and progressive, the environment in the pathogenic microbial biofilm (plaque) becomes increasingly acidic. Aciduric bacteria then thrive at the expense of other benign bacteria. The growth of acidic bacteria is primarily characterized by their ability to dissolve tooth minerals, facilitated by bacterial acids such as lactic, formic, acetic, and propionic acids [20]. The causal bacteria are categorized into two main genera: *Streptococcus* (primarily *Streptococcus sobrinus* and *Streptococcus mutans*) and *Lactobacillus* [19,20].

Periodontitis is characterized by both proinflammatory events and infection, resulting in clinical attachment loss, gingival bleeding, the presence of periodontal pocketing, and radiographically assessed alveolar bone loss. Poor oral hygiene leads to plaque accumulation at and in the subgingival margin, which is the primary cause of periodontal disease [21]. In older adults, periodontal disease may be causally associated with aspiration pneumonia, often leading to significant morbidity and mortality [22]. Eventually, periodontitis can lead to tooth loss, negatively impacting chewing function.

It is now known that plant foods, including herbs, grains, and fruits, contain a vast variety of biologically active compounds that promote health and well-being. There should be a greater emphasis on appropriate dietary phytochemical intake, as it has several advantages. For example, a diet rich in phytochemicals can decrease periodontal diseases, dental caries, and plaque. Certain herb-based foods and beverages may deserve special attention in the diet due to their unique phytochemical composition and medicinal properties. Herbal extracts and their products could serve as alternatives to synthetic compounds for controlling oral diseases. The potential of dietary polyphenols to evoke therapeutic effects may be mediated by alterations in the gut microbiota and gut microbiome composition, leading to improvements in human health [23].

This review delves into our current understanding of the roles that polyphenols, terpenes, and terpenoids, which originate from medicinal plants, play in maintaining oral health. The dietary aspects of polyphenols concerning oral health [24] and general health [14,25,26] have been discussed elsewhere.

The present review synthesizes two key areas. First, we explore the key phytochemicals (polyphenols, terpenes, and terpenoids) present in medicinal plants and their roles in preventing and treating oral and dental diseases and conditions. Our focus is specifically on medicinal plants, rather than foods or processed foods. Second, this review provides an up-to-date overview of the health-promoting benefits of these bioactive compounds, with a special focus on their anti-inflammatory, antioxidant, antimicrobial, anti-allergenic, antimutagenic, and anticarcinogenic properties. This dual focus sets our review apart, shedding light on the multifaceted potential of these natural compounds in oral healthcare.

## 2 Methods

The literature on phytochemicals and their applications in oral health and dental medicine is extensive. We utilized various international databases of online traditional media, including Science Direct, Web of Science, Scopus, Springer, PubMed, Google Scholar, Mendeley, ACS, Taylor, and Kudos, employing search terms individually or in combination. The analysis consisted of peer-reviewed journal articles, book chapters, and authored books. However, our examination of these media discourses has some limitations, such as a focus on print media and the inclusion of only English-language articles.

To determine relevant publications, we reviewed abstracts for general suitability and critically evaluated the experimental methodology and findings of full-text articles. For each subfield, we used pertinent terms as either the search term in the title module or the entire article to retrieve documents. Initially, we tested numerous search terms to identify those that yielded articles relevant to our topic and met our inclusion criteria. Specifically, when conducting research on oral and dental-related literature pertaining to herbs and their bioactive compounds, we employed a search strategy that involved the inclusion of either “oral,” “dental,” or both as search terms. Consequently, for Section 7 of this article, the four primary search terms utilized were “herb’s English name,” “Latin name,” “oral,” and “dental.” These terms were combined with one or more of the following terms: “antibacterial,” “bioactive,” “flavonoid,” “phenolic,” “polyphenol,” “phytochemical,” “tannin,” “terpene,” or “terpenoid.” We then applied filters, including chronological values (i.e., year) and descriptive fields (i.e., title), to narrow down the number of articles found and succinctly summarize their findings in our manuscript. We preferred to cite articles published within the last 6 years (2019–2024) while also incorporating numerous significant older articles that laid the groundwork for herbs, bioactive compounds, and applied health literacy. Additionally, we referred to older articles in instances where there was limited availability of recent publications with substantial scientific evidence on the subject.

## 3 Phenolic compounds

**Figure 1:**
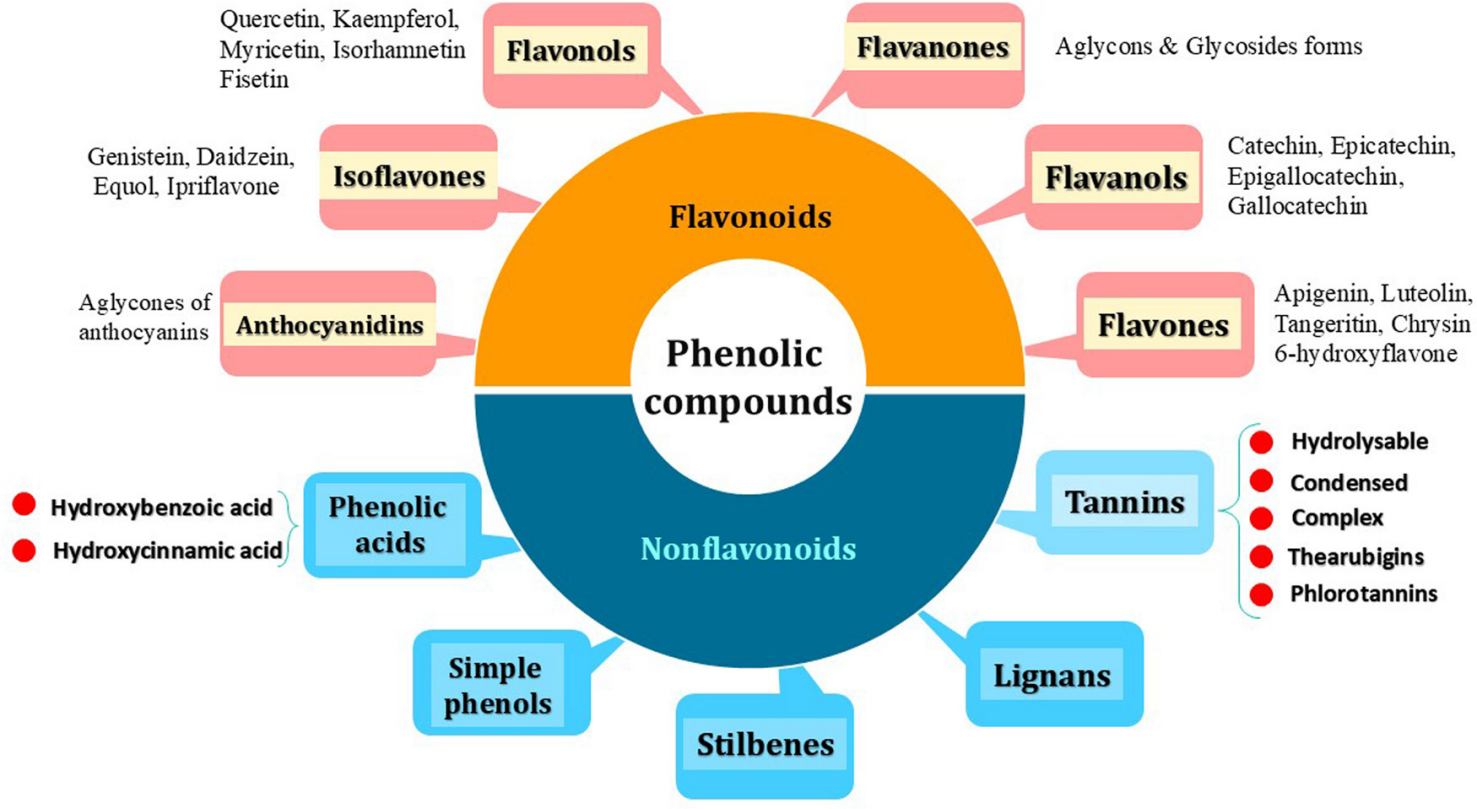
Classification of phenolic compounds most of which are known to be polyphenols.

Phenolic compounds are a diverse and large group of secondary metabolites (>8,000) that include many different families of aromatic compounds in the plant kingdom (Figure 1). As shown in Table 1, simple phenols and polyphenols are the general terms used to categorize them based on chemical nomenclature, which considers factors such as the number of carbons and the structure of the aromatic ring [27]. These compounds are primarily synthesized through the shikimic acid pathway and phenylpropanoid metabolism.

**Table 1:** Classification of phenolic compounds (adapted from Harborne [27])

Structure	Phenolic class
C6	Simple phenolics
C6–C1	Phenolic acids and related compounds
C6–C2	Acetophenones and phenylacetic acids
C6–C3	Cinnamic acids, cinnamyl aldehydes, cinnamyl alcohols
C6–C3	Coumarins, isocoumarins, and chromones
C15	Chalcones, aurones, dihydrochalcones
C15	Flavans
C15	Flavones
C15	Flavanones
C15	Flavanonols
C15	Anthocyanidins
C15	Anthocyanins
C30	Biflavonyls
C6–C1–C6, C6–C2–C6	Benzophenones, xanthones, stilbenes
C6, C10, C14	Quinones
C18	Betacyanins
Lignans, neolignans	Dimers or oligomers
Lignin	Polymers
Tannins	Oligomers or polymers
Phlobaphenes	Polymers

### 3.1 Simple phenols

Simple phenols are compounds that have one or more hydroxyl groups (OH) attached to an aromatic ring (C6). Phenol, resorcinol, pyrocatechol, and phloroglucinol are simple phenols. While these phenolic compounds are not commonly found in plant constituents, resorcinol and phloroglucinol can be combined with cinnamic acids to create various plant flavonoids. Phenol (hydroxybenzene) is rarely present in phytochemicals [27].

### 3.2 Polyphenols

Polyphenols are complex substances characterized by having two or more phenolic rings. Many polyphenols are found in conjugated form, covalently linked with one or more sugar moieties. This linkage can occur through *O*-glycosidic bonds or, less commonly, through *C*-glycosidic bonds. Additionally, polyphenols can form ester bonds with organic acids, such as chlorogenic acids and green tea catechins [28].

In most cases, the term “polyphenols” is used as a general term to encompass all plant-derived phenolic compounds, including both simple phenols and polyphenols, as outlined in this review. Polyphenols are broadly classified into two main groups, flavonoids, and nonflavonoids, depending on the complexity of their chemical structure, specifically, the presence or absence of the C6–C3–C6 backbone [29]. Furthermore, polyphenols are further subdivided into five major categories based on their chemical backbones: phenolic acids, lignans, stilbenes, tannins, and flavonoids, as illustrated in Figure 1.

#### 3.2.1 Flavonoids

A phenyl benzopyran backbone (C6–C3–C6) and two benzene rings (or aromatic rings: A and B) linked via a heterocyclic pyran ring (ring C) are found in the chemical structure of flavonoids. Over 6,000 flavonoid compounds have already been identified [30], which differ in the number and position of the hydroxyl groups, as well as the extent of alkylation and/or glycosylation. These compounds can be classified into eight major groups, each differing in the pyran ring. These groups include flavones, flavanones, isoflavones, flavonols, dihydroflavonols, flavan-3-ols, chalcones, and anthocyanidins. For example, flavonols such as quercetin and kaempferol have a 3-hydroxy pyran-4-one group on the C ring, while flavanols such as catechins have only a 3-hydroxyl group on the C ring [31].

Flavonoids play important roles in preventing various diseases in humans and are generally considered health-promoting factors. They are known for their strong radical scavenging and antioxidant properties. Among flavonoids, flavonols, including quercetin, kaempferol, and myricetin, are considered some of the most important. Quercetin, in particular, is the most abundant flavonol compound in the human diet [32] and can be commonly found in fruits, vegetables, herbs, and spices. Onions, leeks, broccoli, curly kale, apples, and berries are among the richest dietary sources of flavonols [33]. However, the relative importance of these food sources varies depending on the country of origin and, to a lesser extent, the environmental conditions during plant growth. For instance, apples, onions, and tea are the predominant dietary sources of flavonols in the United States, Denmark, and the Netherlands, respectively [34].

#### 3.2.2 Nonflavonoid polyphenols

Nonflavonoids consist of one or two aromatic rings and can be categorized as phenolic acids, hydrolyzable tannins, and stilbenes, which have more complex structures. This group of polyphenols includes compounds with multiple phenol rings, such as tannins, stilbenes, lignans, and phenolic acids. Among them, tannins are the most intricate polyphenol structures due to their polymeric nature. On the other hand, catechins are considered monomeric polyphenols composed of flavan-3-ol monomers and are classified within the flavonoid class.

##### 3.2.2.1 Phenolic acids

Phenolic acids, also known as phenol carboxylic acids, are characterized by a single phenolic ring that is conjugated to a carboxylic acid through a short hydrocarbon chain. Based on the number of carbon units in the lateral chain attached to the phenolic ring, phenolic acids can be classified into C6–C3, C6–C2, and C6–C1 compounds [35]. The two major groups of phenolic acids are hydroxybenzoic acids (C6–C1) and hydroxycinnamic acids (C6–C3), with C6–C3 being the most prevalent in plant-based foods and beverages such as herbs, vegetables, fruits, tea, and coffee. The third group of phenolic acids is referred to as hydroxyphenyl acids, which include acetic, propanoic, and pentenoic acids.

The distinction between simple phenols (C6) and polyphenols is unclear, and phenolic compounds cannot be strictly classified as simple phenols or polyphenols. Some experts consider them separate entities, while others include phenolic acids in the broad category of polyphenols. Dietitians and nutritionists often refer to phenolic acids as “dietary polyphenols.” The most commonly known hydroxycinnamic acids (C6–C3) include *ρ*-coumaric acid, ferulic acid, sinapic acid, caffeic acid, chlorogenic acid, and rosmarinic acid. Gallic, *ρ*-hydroxybenzoic, vanillic, syringic, and ellagic acids are common examples of hydroxybenzoic acids (C6–C1). Phenolic acids can also be classified into two subgroups: free (soluble) and bound (insoluble) phenolic acids [36].

##### 3.2.2.2 Tannins

Tannins are polyphenolic compounds that constitute the most abundant phytochemicals after lignins. They are classified into two major categories: hydrolyzable and nonhydrolyzable [37,38,39]. Nonhydrolysable tannins are also called condensed tannins (CTs) or proanthocyanidins (PACs). Hydrolyzable tannins structurally contain several phenolic acid molecules and are classified based on hydrolyzed gallic acid and ellagic acid as gallotannins and ellagitannins, respectively. CTs are oligomers and polymers of flavan subunits. A crucial characteristic of tannins is their ability to interact with proteins, denaturing them in vertebrate herbivores. This interaction forms the basis of the astringent, ROS-reducing, antimutagenic, antiviral, and antimicrobial properties of tannins.

Astringency and bitterness are organoleptic characteristics widely linked to tannin compounds. The sensation of dryness, puckering, shrinking, and roughening in the mouth is known as astringency. At high concentrations, the astringency of CTs may partially mask the perception of bitterness. Astringency can occur following the consumption of plant-based foods and beverages, such as strong tea, berries, and unripened fruits. The ability of tannin compounds to bind and precipitate proteins, such as proline-rich salivary proteins and glycoproteins, is thought to be the primary driver of astringency. It reduces the coating and lubrication of oral surfaces, causing friction and, at the same time, enables some astringent compounds to interact with oral mucosal substrates [40]. Recently, there has been speculation on whether astringency might be useful for oral health [41]. These authors contend that tannins and related polyphenols directly bind to exposed oral epithelial cells, activating the Piezo2 ion channel as another probable mechanism of astringency perception. Finally, astringency may improve the bioavailability and bioaccessibility of astringent polyphenols, leading to stronger anti-inflammatory, antioxidant, anti-aging, and anticancer effects.

## 4 Terpenes and terpenoids

Terpenes, the largest and most diverse class of secondary metabolites, are composed of five-carbon isoprene units that can be combined in numerous ways and often contain oxygen. They are diversely present in plants, with over 80,000 known terpenes and terpenoids found in the wild [13]. The varying levels and combinations of these compounds give each plant unique characteristics. Notably, terpenes play a role in the pleasant smell, spicy taste, and biological effects of plants. Pine trees, lemon, lavender, and peppermint are examples of plants with particularly noticeable terpene contents [42]. Terpenes are basic hydrocarbons, whereas terpenoids constitute an altered group of terpenes characterized by distinct functional groups and the relocation or elimination of oxidized methyl groups at different locations [43]. Terpenoids are typically divided into monoterpenes, sesquiterpenes, diterpenes, sesterpenes, and triterpenes depending on their carbon units. Various plants exhibit unique terpene profiles, and certain types of terpenes are exclusive to specific species.

Terpenes and terpenoids are categorized based on the isoprene rule, determined by the number and arrangement of carbon atoms in isoprene units. The classification includes hemiterpenes (C_5_H_8_), monoterpenes (C_10_H_16_), sesquiterpenes (C_15_H_24_), diterpenes (C_20_H_32_), sesterterpenes (C_25_H_40_), triterpenes (C_30_H_48_), tetraterpenes (C_40_H_64_), and polyterpenes (>8 isoprene units). It has been demonstrated that oxygenated terpenes (phenolic terpenoids), such as carvacrol, *ρ*-cymene, and thymol, display stronger antimicrobial activity than hydrocarbon terpenes, such as camphene, R-(–)-limonene, terpinene, and (+)-*α*-pinene [44]. Each plant can contain numerous terpenes, but certain terpenes are commonly found in high quantities across multiple plant species. The following are some of the most important terpenoid compounds found in plants: artemisinin, β-carotene, β-caryophyllene, *ρ*-cymene, farnesol, germacrene (germacrene a and d), humulene (α-caryophyllene), limonene, linalool, lupeol, lycopene, menthol, myrcene, ocimene, α-pinene, β-pinene, squalene, taxol, and terpinolene. Table 2 presents the terpenes, their type, and the plants rich in these compounds, focusing on those relevant to our theme of oral health-benefiting plants.

**Table 2:** The terpenes, their type, and the plants rich in these compounds

Terpene compound	Terpene type	Medicinal herbs contain the compound
Artemisinin	Sesquiterpene (C_15_)	Mugwort, levant wormseed, sweet wormwood, tarragon, wormwood
β-Carotene	Tetraterpene (C_40_)	A natural precursor of vitamin A found in parsley, fenugreek, amaranth, coriander, dandelion, cilantro
β-Caryophyllene	Sesquiterpene (C_15_)	Cloves, cinnamon, and black pepper
*ρ*-Cymene	Monoterpene (C_10_)	Oregano, thyme, mint
Farnesol	Sesquiterpene (C_15_)	Chamomile, rose, lemongrass, ginger
Germacrene (germacrene A and D)	Sesquiterpene (C_15_)	Arnica and yarrow
Humulene (α-caryophyllene)	Sesquiterpene (C_15_)	Hops, sage, ginseng
Limonene	Monoterpene (C_10_)	Citrus fruits: lemon, orange, grapefruit
Linalool	Monoterpene (C_10_)	It has soothing qualities and a distinct herbal aroma, is found in lavender and rosewood
Lupeol	Triterpene (C_30_)	Aloe vera, neem, basil, ginger, turmeric
Lycopene	Tetraterpene (C_40_)	Tomato, watermelon, apricot
Menthol	Monoterpenes (C_10_)	Mint, peppermint, spearmint, thyme, eucalyptus
Myrcene	Monoterpene (C_10_)	Its name derives from *Myrcia sphaerocarpa* but is also present in lemongrass, hops, and mangos
Ocimene	Monoterpene (C_10_)	Mint, parsley, orchids
*α*-Pinene	Monoterpene (C_10_)	Pine, rosemary, eucalyptus, juniper, cumin, sage, frankincense, lavender, thyme,
*β*-Pinene	Monoterpene (C_10_)	Rosemary, sage, coriander, cumin, nutmeg, thyme
Squalene	Triterpene (C_30_)	Amaranth, olive oil, palm oil, avocado
Taxol	Diterpene (C_20_)	Yew (*Taxus* spp.)
Terpinolene	Monoterpene (C_10_)	Hemp, nutmeg, cumin, and apples
Tocopherols (α-, β-, γ-, and δ-tocopherol)	Diterpene (C_20_)	Avocados, spinach, kale, broccoli, vegetable oils (safflower, sunflower, olive, soybean, corn oils)

## 5 Basis of the antioxidant activity of phenolic compounds

Phenols are antioxidants found in most plant-derived products. *In vitro* studies have shown that polyphenols exhibit robust antioxidant activity and are capable of effectively scavenging a wide array of reactive oxygen, nitrogen, and chlorine species, including hydroxyl radicals, superoxide anions, hypochlorous acid, peroxyl radicals, and peroxynitrous acid [45]. Various mechanisms have been suggested to explain the antioxidant activity of phenolic compounds, including the suppression of free radicals through hydrogen atom donation, the reduction of these reactive molecules via electron transfer, and the chelation of transition metals. The antioxidant effectiveness of phenolic compounds greatly relies on the presence of hydroxyl (−OH) groups and the degree of methoxylation [46].

The oral mucosa is constantly exposed to oxidative stress caused by environmental and food factors. Polyphenols, when in direct contact with the oral cavity, can help prevent various oral diseases, ranging from infections to cancers, thanks to their antioxidant and antimicrobial properties [47]. Polyphenols can play a role in reducing the risk of oral cancer by scavenging free radicals. They also assist in preventing oxidative stress and DNA damage, controlling the metabolism of carcinogens, and hindering the formation of DNA adducts [48].

Polyphenols are known to have greater antioxidant (antiradical) activity than simple phenols [49]. Enhanced antioxidants may be those that undergo oxidation more readily. On the other hand, certain small phenols/polyphenols that easily undergo oxidation may exhibit pro-oxidant properties, particularly at higher concentrations, rendering them more susceptible to pro-oxidant factors than larger molecular weight compounds [50].

## 6 Herbs and oral health care

Given the challenge of antibiotic resistance in the treatment of periodontal diseases, host immune modulation using phytocompounds represents one of the exciting new developments for treatment and prevention. It is well known that good oral hygiene and regular dental care are important practices that support both oral and general health. Medicinal plants that are rich in antioxidant, anti-inflammatory, and antimicrobial agents are good candidates for application in dentistry. Some medicinal plants with the aforementioned features include but are not limited to aloe vera, anise, black cumin, bloodroot, caraway, chamomile, cinnamon, clove, coriander, cumin, emblic, eucalyptus, fenugreek, garlic, ginger, green tea, common guava, miswak, myrrh, neem, mint (peppermint, spearmint, and cornmint), rosemary, sage, thyme, tulsi, turmeric, and yarrow (see Table 3 for a more extensive list).

The essential oils or extracts of plants derived from either roots or aboveground organs (such as stems, bark, rhizomes, leaves, flowers, fruits, and seeds) are frequently incorporated into mouthwash and toothpaste formulations as oral care ingredients. The extracts obtained from either infusions or decoctions are administered orally, for example, as a mouth rinse or for ingestion, to promote dental and oral health.

### 6.1 Herbal mouthwash and toothpaste

A variety of mouthwashes are used today for several reasons, including freshening breath, preventing dental caries, reducing plaque and gingivitis, and slowing the formation of dental calculus. Currently, herbal mouthwashes have gained popularity due to their natural origin, fewer side effects, and lower toxicity. These mouthwashes contain oral health-related ingredients, which can be categorized into three main materials: fluoride compounds, antimicrobial agents, and plant extracts. Oral hygiene solutions that consist of botanical ingredients have been proven effective in reducing tooth plaque and gingivitis and in alkalizing saliva to change its pH [24,51,52,53]. A clinical assessment of 15-day durations using 300 participants conducted by Sharma et al. [54] showed that a mouthwash containing tulsi is less effective as a mouth rinse than chlorhexidine or hydrogen peroxide. However, tulsi was found to effectively reduce plaque, halitosis, and gingivitis.

Toothbrushing is known as an oral care strategy that effectively removes dental plaque and prevents periodontal disease by inhibiting the growth of plaque and bacterial adherence to the pellicle. Dental plaque (biofilm) is now recognized as a major cause of the two most prominent dental diseases: periodontal and caries disease [55]. Laboratory and clinical research indicate that toothpaste containing botanical ingredients can reduce dental gingivitis, plaque, tooth staining, and dental calculus [56,57,58,59,60]. A recent systematic review and meta-analysis of randomized controlled clinical trials by Adam et al. [61] revealed that miswak (*Salvadora persica*)-based toothpaste has antigingivitis and antiplaque effects comparable to those of nonherbal toothpaste. However, few studies have explored the feasibility and efficacy of a toothpaste containing multiple botanical ingredients. Jayashankar et al. [62] conducted a double-blind randomized clinical trial using 60 participants (test *n* = 30, control *n* = 30) to study the efficacy of a toothpaste containing a mixture of herbal ingredients from nine plants (*Acacia chundra* (Roxb. ex Rottler) Maslin heartwood, *Adhatoda vasica* L. leaves, *Mimusops elengi* L. barks, *Piper nigrum* L. seeds, *Pongamia pinnata* (L.) Merr. roots, *Quercus infectoria* Oliv. galls, *Syzygium aromaticum* (L.) Merr. & Perry flowers, *Terminalia chebula* Retz. fruits, and *Zingiber officinale* Roscoe rhizomes) in reducing plaque, gingivitis, and bleeding compared to a control (nonherbal toothpaste). Cinnamon (*Cinnamomum zeylanicum* Blume) and oregano (*Origanum vulgare* L.) essential oils, when incorporated into herbal toothpaste formulations, exhibited antibacterial activity against *S. mutans* [63].

### 6.2 Medicinal plants as antimicrobial agents in dental caries

Dental caries is a multifactorial disease primarily caused by cariogenic bacteria commonly found in the oral cavity. It affects the hard tissues of the tooth by promoting the development of biofilm and plaque, which leads to surface roughness, demineralization, cavitation with pulp compromise, abscess, and swelling.

The healing properties of herbs are attributed to various bioactive compounds (see Tables 3 and 4). Phenolic compounds, such as flavonoids and tannins, are abundant and phytochemically important components of botanical drugs [4,64]. In both *in vivo* and *in vitro* studies, various herbal essential oils and isolated major compounds have demonstrated antimicrobial activity against a wide range of human pathogenic microorganisms, including viruses, bacteria, fungi, and protozoans. Notably, isolated phenolic compounds such as eugenol, thymol, and carvacrol exhibit strong antimicrobial effects [4].

**Table 3:** Medicinal plants used for oral and dental health

Species	Common name	Preparation/uses	Oral/dental application
*Aloe vera*	Aloe vera	Leaf, stem extracts	Dental abscess; oral antimicrobial [70]
*Arnica montana*	Arnica	Flower heads (capitula), oil, extract	Periodontal tissue inflammation; postsurgical pain [75,76]
*Achillea millefolium*	Yarrow	Leaf oil, extract	Teeth cleaning/braces, toothache [171]
*Allium sativum*	Garlic	Bulb oil, extract	Oral antimicrobial [208]
*Anthemis nobilis*/*Chamomila recutita*	Chamomile	Leaf/flower heads (capitula); oil; extract; mouthwash	Treatment of mucosa, oral cavity, and skin inflammations [78,79]
*Azadirachta indica*	Neem	Chewing stick; mouthwash	Antiinflammatory/antimicrobial [209]
*Calendula officinalis*	Marigold	Flower head/oil infusion	Oral wound healing, anti-inflammatory, antimicrobial [210]
*Capsicum frutescens*	Wild pepper	Fruit; fruit juice	Oral mucositis pain; secondary to chemotherapy and radiation therapy [211]
*Chondrus crispus*	Irish moss	Seeds, gum, mucilage	Antimicrobial and antibiofilm: prevent biofilm formation in dental plaque [212]
*Camellia sinensis*	Green tea	Leaf oil, extract	Dental caries, periodontal disease, and halitosis [104]
*Carum carvi*	Caraway	Dried seed and fruit oil	Antimicrobial and antibiofilm [213]
*Chrysanthemum parthenium*	Feverfew	Leaf	Toothache [214]
*Cinnamomum zeylanicum*	Cinnamon	Bark; powdered bark infusion	Antimicrobial and anti-inflammatory properties [80]
*Commiphora myrrha*	Myrrh	Bark resin tincture application/gargle	Mouthwash: oral hygiene, antiplaque, and treating gingivitis [215]
*Coriandrum sativum*	Coriander	Leaf; seed oil, extract	Mouthwash; oral antimicrobial [216]
*Curcuma longa*	Turmeric	Rhizome, oil, extract	Various oral lesions and conditions [217]
*Echinacea angustifolia*	Coneflower	Flower	Toothache; oral anti-inflammatory [218]
*Eucalyptus globulus*	Eucalyptus	Leaf extract	Oral antimicrobial [219]
*Foeniculum vulgare*	Fennel	Seed oil; fruit ingestion	Antibacterial and antibiofilm [220]
*Ganoderma lucidum*	Reishi	Fruit body, mycelia	Antimicrobial [203]
*Hydrastis canadensis*	Goldenseal	Rhizome and root infusion	Oral antimicrobial [221]
*Lentinus edodes*	Shiitake	Fruit body, mouthwash	Antimicrobial, antiplaque [199,201]
*Mentha piperita*	Pepermint	Aerial part (leaf/flower) oil	Toothache; mouthwash, toothpaste [4]
*Mentha spicata*	Spearmint	Aerial part (leaf/flower) oil	Toothache; mouthwash, toothpaste [4]
*Mentha arvensis*	Cornmint	Aerial part (leaf/flower) oil	Toothache; mouthwash, toothpaste [4]
*Nigella sativa*	Black cummin	Seed oil, extract	Oral antimicrobial [222]
*Ocimum sanctum*	Tulsi	Leaf oil, extract	Teeth cleaning; oral antibiotic [166]
*Origanum vulgare*	Oregano	Leaf oil; leaf decoction	Oral antimicrobial; mouthwash [132]
*Phyllanthus emblica*	Emblic	Fruit	Oral antimicrobial [223]
*Pimpinella anisum*	Anise or aniseed	Seed extract	Oral antimicrobial [224]
*Plantago major*	Plantain	Leaf; seed	Oral antibacterial [225]
*Psidium guajava*	Guava	Leaf, bark, fruit oil/extract	Oral antimicrobial [226]
*Rosmarinus officinalis*	Rosemary	Leaf, oil, extract: mouthrinse	Antimicrobial, toothpaste [136]
*Rubus fruticosus*	Blackberry	Fruit extract	Mouthwash to relieve mouth ulcers and gum inflammation
*Salvia officinalis*	Sage	Leaf, oil, extract; mouthwash	Anti-inflammatory, antimicrobial [139]
*Salvadora persica*	Miswak	Chewing gum; oil, extract, tooth powder: mouthwash	Anti-inflammatory, antimicrobial [110,116]
*Sanguinaria canadensis*	Bloodroot	Rhizome extract	Oral antimicrobial; dentifrice [227]
*Simmondsia chinensis*	Jojoba	Flower and seed oil	Mouthrinse, dental varnish [228]
*Stevia rebaudiana*	Stevia	Leaf extract; mouthrinse	Antimicrobial, anti-plaque [146]
*Syzygium aromaticum*	Clove	Powdered clove ingestion; dried flower bud oil	Antimicrobial; toothpastes, mouthwash [84,229]
*Thymus vulgaris*	Thyme	Aerial part (leaf/flower) oil	Antimicrobial, toothpaste [4]
*Trigonella foenum-graecum*	*fenugreek*	Seed oil, extract	Oral antimicrobial [230]
*Vaccinium macrocarpon*	Cranberry	Fruit	Antibacterial, antibiofilm and anti-inflammatory [11]
*Zanthoxylum americanum*	Prickly ash	Bark decoction	Toothache; antimicrobial
*Zingiber officinale*	Ginger	Rhizomes oil; mouthwash	Oral anti-inflammatory, antimicrobial [94]

**Table 4:** Description and structure of key polyphenols significantly impacting dentistry and oral health

Compound/description	Chemical structure (all from ChemIDplus)	Herb source (major component)
Eugenol, a phenylpropanoid, is extensively utilized in dentistry to manage toothaches and pulpitis [84]	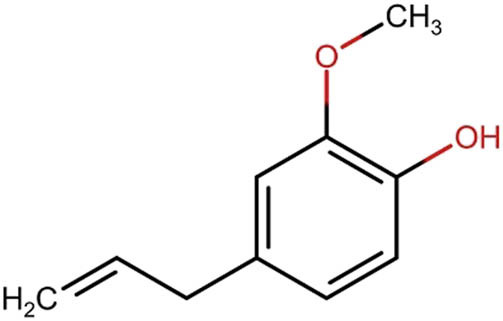	Clove (*Syzygium aromaticum*); cinnamon (*Cinnamomum zeylanicum*)
Catechin, a polyphenolic flavonoid, is beneficial to periodontal and gingival health by inhibiting bacterial biofilm formation, attenuating host inflammatory responses, and demonstrating antibacterial attributes against periodontal pathogens by inhibiting their proteolytic enzymes [11]	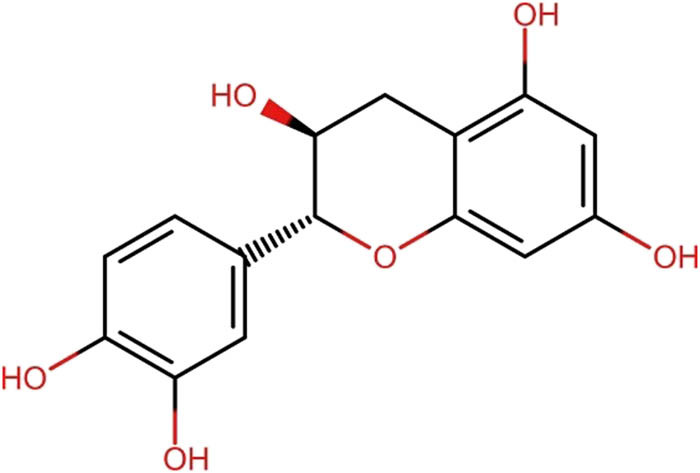	Cranberry (*Vaccinium macrocarpon*)
Epigallocatechin-3-gallate, categorized as a catechin, is associated with improved oral health and a reduced likelihood of developing cavities when included in animal or human diet [99,100]	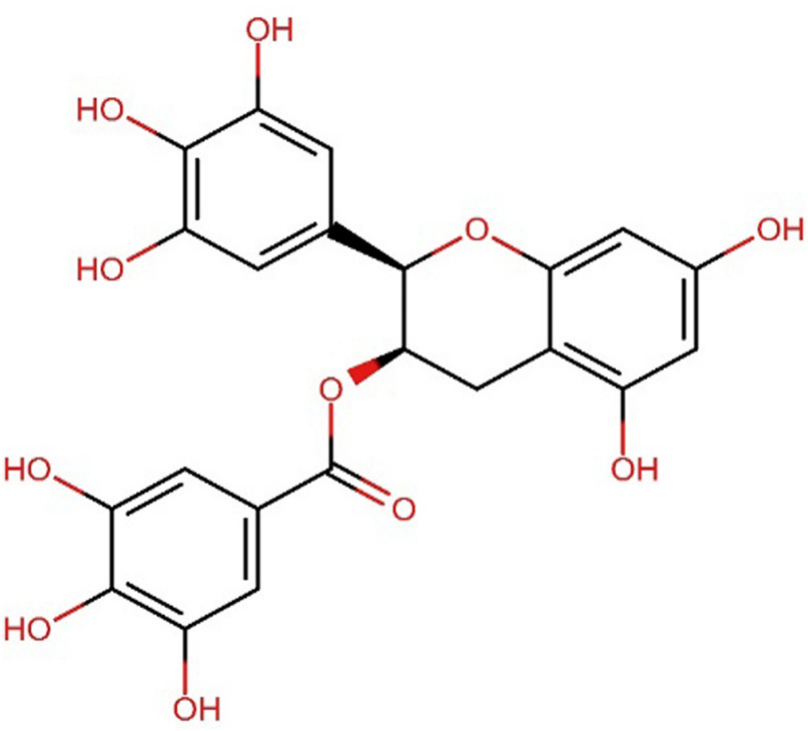	Green tea (*Camellia sinensis*)
Gingerol (6-gingerol), a polyphenolic compound, serves as a potent disinfectant for root canals [90] and effectively modulates the oral microbiota [91]	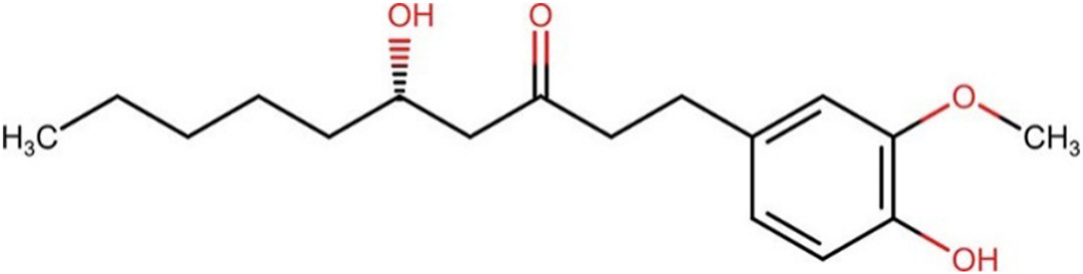	Ginger (*Zingiber officinale*)
Thymol serves as an antibacterial, analgesic, and oral cavity disinfectant. As a result, it is employed in the manufacturing of dental and oral care products, including hygiene items, and as a flavor-enhancing component in toothpaste, chewing gum, and pharmaceuticals [4]	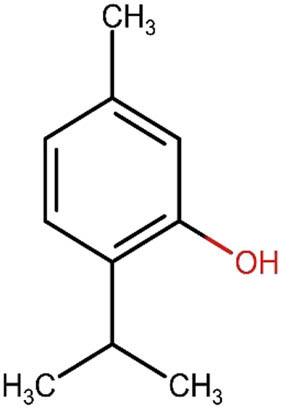	Thyme (*Thymus vulgaris*); Oregano (*Origanum vulgare*)
Curcumin, a vibrant yellow polyphenolic pigment, possesses robust anti-inflammatory and antioxidant properties that could be beneficial in addressing or preventing oral health conditions [157]	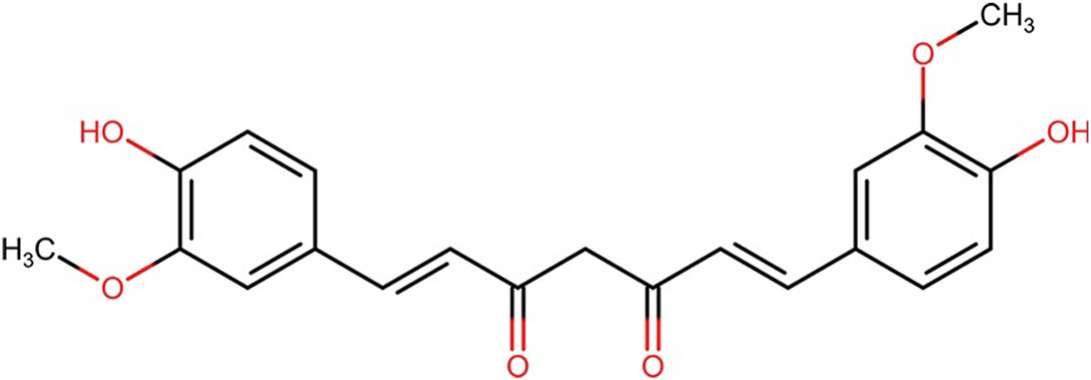	Turmeric (*Curcuma longa*)

The use of botanical drugs for the treatment of dental caries, gingivitis, and periodontitis has gained significant attention among dental professionals and patients in recent years. In an *in vitro* study conducted by Besra and Kumar [65], the antimicrobial activity of 20 medicinal plant extracts against bacterial pathogens associated with dental caries was evaluated. The findings showed that the following plants demonstrated significant antimicrobial activity against the cariogenic pathogens *S. mutans*, *Lactobacillus acidophilus*, *Achyranthes aspera* L. (root), *Azadirachta indica* A. Juss (leaves), *Nigella sativa* L. (seeds), *Psidium guajava* L. (leaves), and *S. aromaticum* (bud).

Moreover, an interview survey conducted among traditional healers and folk practitioners in Baghdad, Iraq, revealed that clove (*S. aromaticum*), green tea (*Camellia sinensis*), and mint (*Mentha* spp.) were the three most frequently employed botanical drugs for addressing toothaches, canker sores, oral ulcers, and homemade mouthwash preparations [66].

Eugenol is extensively utilized in dental procedures due to its significant antibacterial and anesthetic qualities. It serves as the primary compound found in dianthus oil. Eugenol is commonly incorporated into root canal sealers (such as Endomethasone and Caryosan), temporary fillings and pastes utilized for direct pulp capping (such as Caryosan and zinc oxide). Additionally, it can be employed for disinfecting tooth canals during the treatment of pulp necrosis or as a precipitant for dentin impregnation with silver nitrate [67].

Table 4 illustrates the structures of key phenolic compounds that play a significant role in dentistry and oral health.

## 7 Medicinal plants used in oral health and dentistry

### 7.1 Aloe vera [*Aloe barbadensis* Miller (Liliaceae)]

The gel extracted from the chlorenchyma and parenchyma tissues of *Aloe vera* leaves contains a wide variety of bioactive compounds that possess significant antioxidant, anti-inflammatory, antimicrobial, and angiogenic activities [68]. Namiranian and Serino [69] carried out a randomized, double-blind, intraindividual, and controlled clinical study. In this study, they examined the effectiveness of two types of dentifrice: one containing fluoride (used as a control) and the other containing aloe vera. The study involved two groups of patients and spanned a 30-day duration. Similarly, both groups showed a significant reduction in gingivitis and plaque. In another study, a toothpaste containing *A. vera* and *Fragaria vesca* L. (wild strawberry) exhibited antimicrobial activity against *S. mutans*, which is a member of the oral microbiome and a principal cause of dental caries [70].

The survival of *S. mutans* depends strictly on a dynamic biofilm ecosystem, namely, dental plaque. *A. vera* has been found to be effective in treating oral ulcers and oral lichen planus, as it promotes healing and immunomodulation [71]. Furthermore, an *A. vera*-containing mouthwash significantly reduced gingivitis and plaque, although it was still significantly less effective than a chlorhexidine-containing mouthwash [72].

### 7.2 Arnica [*Arnica montana* L. (Asteraceae)]

The flower heads (capitula) of arnica are used in herbal medicine. The essential oil of flower capitula comprises polyphenolics, such as chlorogenic acid, 3,5-dicaffeoylquinic acid, 1-methoxyoxaloyl 3,5-dicaffeoylquinic acid, quercetin, and kaempferol, as well as terpenoids like alpha-pinene, *ρ*-cymene, germacrene D, *β*-pinene, and spathulenol. These compounds are the major bioactive phytochemicals found in the essential oil of flower capitula [73,74].

Dried arnica specimens are mainly used for their antioxidant, antiseptic, anticancer, anti-inflammatory, analgesic, antimicrobial, and immunomodulatory activities. However, despite its numerous applications, the use of *A. montana* in dentistry has not been thoroughly explored, possibly due to the lack of evidence-based treatment guidelines for incorporating it into dental practices. Nonetheless, studies have described the potential benefits of arnica in managing and preventing periodontal tissue inflammation due to its anti-inflammatory and antimicrobial properties [75]. In fact, topical *A. montana*-containing oral homeopathic solutions have been shown to significantly reduce postsurgical pain [76].

### 7.3 Chamomile [*Matricaria chamomilla* L. syn. *Chamomilla recutita* (L.) Rauschert (Asteraceae)]

Flower heads (capitula) of chamomile are extensively employed in the preparation of tea, providing numerous health and medicinal benefits. The most imperative constituents relating to chamomile’s beneficial attributes are flavonoids (luteolin, apigenin, and quercetin) and terpenoids (bisabolol and chamazulene). It has antioxidant, wound healing, spasmolytic, anxiolytic, anti-inflammatory, antimicrobial, analgesic, antinociceptive, and mild sedative properties. Chamomile and external/internal infusions are used in the treatment of mucosa, oral cavity, and skin inflammation [77,78]. It is effective as a mouthwash for treating gingivitis and periodontal disease due to its anti-inflammatory properties. Chamomile-containing mouthwash effectively reduces gingivitis and dental plaque [79].

### 7.4 Cinnamon [*C. zeylanicum* Blume (Lauraceae)]

Cinnamon bark is commonly used as a spice and herb with a wide range of applications in perfumery, beverages, flavoring, and medicines. Cinnamon essential oil, along with its extracts and major constituents such as cinnamaldehyde, eugenol, linalool, and *β*-caryophyllene, has shown significant antimicrobial properties against oral microbiota. As a result, it can be used to prevent caries and periodontal disease, as well as to treat oral candidiasis during endodontic treatment [80].

In addition to its antimicrobial properties, cinnamon also possesses antioxidative and anti-inflammatory properties [81]. It has been widely incorporated into dental and oral care products such as mouthwash, toothpastes, dental gel, endodontic irrigants, and mouth fresheners found in mouth rinses [80].

### 7.5 Clove [*S. aromaticum* (L.) Merr. & Perry. Syn. *Eugenia caryophyllata* Thunb. (Myrtaceace)]

The dried aromatic flower buds of clove, an evergreen tree, are used in various ways, such as food, cosmetics, and medicine. Eugenol (4-allyl-2-methoxyphenol, C_10_H_12_O_2_) is the most abundant component of clove essential oil, comprising 45–90% of its composition [82]. Eugenol is known for its antimicrobial, antioxidant, anti-inflammatory, anticancer, and analgesic properties. A variety of mechanisms have been proposed to explain how eugenol inhibits bacterial growth, including disrupting cell membranes by increasing nonspecific permeability and modifying adenosine triphosphate and ion transport [9]. Clove is a popular medicinal plant used for treating toothaches and inflammation in the throat and mouth [83]. Eugenol is commercially obtained from clove flower buds or essential oils. Additionally, eugenol can be synthesized from coniferyl alcohol, which is derived from lignin degradation. Both clove and eugenol can be effectively incorporated into therapeutic strategies such as toothpaste, mouthwashes, topical agents, and local drug delivery systems to attenuate or prevent periodontal diseases [84].

### 7.6 Cranberry [*Vaccinium macrocarpon* Ait. (Ericaceae)]

Cranberry fruit contains a bioactive compound called PACs or flavan-3-ols, which has been found to have beneficial effects in the treatment of periodontal diseases ([85]. Catechin and epicatechin, which are part of the subgroup of polyphenols called flavonoids, are the building blocks of PACs. These compounds exhibit antiadhesive, antimicrobial, and immunomodulatory activities, as well as inhibition of collagenase and proteinase activity [86].

Cranberry’s unique CTs, also known as A-linked type PACs, have greater cariostatic effects compared to other polyphenol-rich plants like tea, which contain B-type PACs [11]. Additionally, this structural variant has been associated with other clinical attributes, such as a reduction in biofilm formation by bacteria, inhibition of proteinase activity in periodontal pathogens, collagenase activity, and associated cytotoxic reactions [87,88]. *In vitro* studies have shown that cranberry fruit constituents have beneficial effects on periodontal and gingival health by inhibiting bacterial biofilm formation, attenuating host inflammatory responses, and exerting antibacterial properties on periopathogens through the inhibition of proteolytic enzymes [11].

### 7.7 Ginger [*Z. officinale* Roscoe (Zingiberaceae)]

Ginger has attracted considerable interest for its antioxidant, antimicrobial, and anti-inflammatory properties due to its phenolic compounds such as gingerol, hydrocarbons, oleoresin, and shogaol [89]. Additionally, it is known for its benefits in oral care and the treatment of dental and oral disorders [89]. Ginger rhizomes are rich in polyphenols, specifically gingerols and shogaols, which exhibit anti-inflammatory and antimicrobial activity. These properties make ginger an effective disinfectant for root canals [90] and for controlling oral microbiota [91].

In a study conducted both *in vitro* and *in vivo* in rats by Hasan et al. [89], the methanolic extract of *Z. officinale* was found to be effective against the virulence attributes of *S. mutans*, suggesting its potential as a prophylactic therapeutic tool for combating dental caries. The key components of ginger that contribute to its antibiofilm, antibacterial, antiviral, antifungal, and analgesic activities are polyphenols (gingerols, shogaol, proanthocyanins, and CTs) and sesquiterpenes (farnesene, zingiberene, and curcumene) [92,93]. Ginger has been extensively researched for its ability to prevent plaque buildup and freshen breath. Using ginger toothpaste and toothpowder aids in maintaining clean teeth and eliminating tartar and cavities [94].

### 7.8 Green tea [*C. sinensis* (L.) O. Kuntze (Theaceae)]

Green tea is known for its high polyphenol content, making it a very healthy beverage. It has various benefits, including antibiofilm [95], anti-gingival inflammation [96], antimicrobial [97], and antioxidant properties [98]. Tea is produced from the leaves and leaf buds of *C. sinensis*. Green tea, which is not fermented, has a higher concentration of catechins than black tea. It contains flavonoids such as epicatechin, epigallocatechin, epicatechin gallate, and epigallocatechin gallate. Among these compounds, epigallocatechin-3-gallate is the main component of green tea [98].

Green tea has been found to have beneficial effects on oral health. It can help reduce the risk of periodontal disease [99,100] by inhibiting collagenase and preventing the adherence of *Porphyromonas gingivalis* to oral epithelial cells [101]. Homemade mouthwash with green tea can also be used to treat aphthous stomatitis, a common form of oral ulceration. Numerous studies have demonstrated the advantages of using green tea for oral health concerns such as dental caries, periodontal disease, and halitosis. These findings suggest that green tea is effective at reducing bacterial activity in the mouth and may also lower the likelihood of developing oral cancer due to its antioxidant properties [102].

Green tea and its main component, epigallocatechin-3-gallate, have protective effects against various diseases and possess antibacterial properties. They can reduce bone loss in osteoporosis and periodontal disease by causing the death of osteoclasts and similar cells [103]. The consumption of green tea is linked to better dental health and a lower risk of cavities [104]. Moreover, it causes less tooth staining than CHX and has been reported to accelerate the healing of mouth ulcers in test groups [105]. Scientific studies indicate that the catechins found in green tea can inhibit the growth of *S. mutans*. The use of polyphenols found in green tea as a mouthwash could be a safe and effective additional treatment for inflammatory periodontal diseases [106]. In our diet, herbal teas provide a valuable source of phytochemicals like flavonoids and other polyphenols.

### 7.9 Mint [*Mentha* spp. L. (Lamiaceae)]

Peppermint (*Mentha piperita*), spearmint (*Mentha spicata*), and cornmint (*Mentha arvensis*) are the most important and commonly cultivated mint species [4]. Peppermint (*Mentha* × *piperita* L.) is an interspecific hybrid mint that is cultivated worldwide. Essential oils and menthol are the most important mint compounds used in medicine and dentistry. The essential oils of peppermint primarily contain monoterpenes such as (–)-menthol, (–)-menthone, methylacetate, menthofuran, (+)-pulegone, isomenthone, 1,8-cineole, linalool, piperitone oxide, and piperitone. Among these compounds, menthol is the most abundant. The phytochemicals and antimicrobial properties of mint (*Mentha* spp.) were recently reviewed by Soleimani et al. [4] and will not be further addressed here.

The steam-distilled essential oils obtained from the aerial parts of peppermint, spearmint, and cornmint are commonly utilized in the production of various food condiments, medicines, mouthwashes, toothpastes, and chewing gums [107]. Among these, commercial menthol and menthone derived from cornmint and peppermint are known as two of the most widely produced and traded herbal remedies around the world. Peppermint possesses cholagogue, spasmolytic, and antidiarrheal properties. In addition to its antimicrobial activity, the fresh and minty flavor, along with its cooling effect, contributes to the creation of a dual-purpose bioactive product. This is why peppermint is extensively used in chewing gums, mouthwashes, and toothpastes [4]. Both peppermint and spearmint are popular choices for herbal infusions, with peppermint ranking among the most widely consumed and favored herbal teas or tisanes. Mint oil has been added to oral care products such as mouthwash, toothpaste, and dental floss due to its effectiveness in eliminating bacteria that cause bad breath and gum disease [108]. Recently, an encapsulated blend of essential oils from four mint species (peppermint, spearmint, bergamot mint, and Japanese mint) was used in oral treatment, leading to a significant reduction in the abundance of several bacterial species associated with periodontal disease and halitosis, including those from the *Streptococcus* and *Actinomyces* genera [109].

### 7.10 Miswak (*S. persica* L.)

Throughout history, miswak has been employed in various ways, including as sticks, extracts, and toothpaste, owing to its powerful medicinal properties [110]. Some sticks made from the branches of the tree are used as an alternative to the common miswak obtained from its roots. Chewing miswak sticks, often called “timeless toothbrushes,” has been a longstanding practice after meals and has been observed throughout history [61,111]. The intention behind this custom was to thoroughly cleanse the teeth and remove any oral biofilms present in the mouth. By combining toothbrushing with the use of miswak chewing sticks, a randomized crossover trial demonstrated improvements in oral hygiene and gingival health, as evidenced by the quantification of *S. mutans* and *Aggregatibacter actinomycetemcomitans* [112]. The effectiveness of this plant is attributed to the presence of benzyl isothiocyanate (BITC), a key component that suppresses acid production and hampers the growth of *S. mutans* and *Streptococcus sanguinis* bacteria [113,114]. In addition to its antimicrobial properties, BITC has antibiofilm [115] and anti-inflammatory [116] effects. In this plant, other important active compounds include salvadourea, salvadorine, trimethylamine, flavonoids, tannins, saponins, sulfur, fluoride, and chloride [117,118]. Most of these compounds provide antibacterial benefits and enhance gingival health. Tannins create a safeguarding barrier on the enamel to prevent caries, and chloride offers protection against the build-up of calculus [119].

This plant species (*S. persica*) has been utilized in various forms, such as chewing gum, probiotic spray, dental cement, chewing sticks, toothpaste, mouthwash, dental varnish, essential oils, and extracts [110]. Studies have demonstrated that miswak exhibits several beneficial effects, including its ability to reduce gingivitis, whiten teeth, preserve orthodontic chains, be compatible with oral cells, prevent tooth decay, promote the healing of gum wounds, and prevent plaque buildup [120]. Although toothpastes and mouth rinses typically clean dentine and oral surfaces, it is possible that various parts of the *S. persica* plant, including its roots, leaves, bark, flowers, and seeds, can provide valuable additional support for dental cleaning and oral hygiene practices. Compared to chlorhexidine and/or placebo mouthwashes, a mouth rinse containing miswak is more effective at reducing plaque and cariogenic bacteria [113]. The extract of *S. persica*, most commonly in the form of mouthwash, has been extensively studied. To obtain more detailed information, we suggest consulting two recent systematic reviews and meta-analyses that explore the effects of *S. persica*-extracted mouthwash on its anti-plaque and anti-caries properties [61,113].

### 7.11 Neem [*A. indica* A. Juss]

The leaves, bark, and seeds of the neem tree are used for the use of herbal insecticides and medicines due to their insecticidal and medicinal properties [6]. The principal phytochemicals of this plant are terpenoid (azadirachtin, gedunin, and nimbolide) and nonterpenoid (polyphenolics) compounds [121]. The antimicrobial activity of neem against prevalent endodontic pathogens, including *Candida albicans*, *Candida parapsilosis*, *Enterococcus faecalis*, *Escherichia coli*, *Klebsiella oxytoca*, *Pseudomonas aeruginosa*, *S. mutans*, and *Staphylococcus aureus*, has been well documented [122,123]. Endodontic irrigation solution obtained from neem and 3% sodium hypochlorite solution were comparably effective in eliminating *E. faecalis* [124]. Moreover, neem twigs are widely used as a source of chewing sticks for dental hygiene by millions of people, particularly in countries where this tree is a native species [6]. This traditional use has rendered the field deeply entrenched in the study of examined *A. indica* products for improving dental hygiene and treating or preventing oral diseases. Notable research advancements include the proposal that *A. indica* mouthwashes are effective alternatives to conventional mouthwashes containing chlorhexidine for reducing gingivitis, plaque, and pain *in vivo* [6,125,126] and against biofilm-forming bacteria (e.g., *P. gingivalis*, *Streptococcus viridans*, and *S. aureus*) as well as *S. mutans* and *C. albicans in vitro* or *ex vivo* [125,127]. Furthermore, the foamability, pH, and organoleptic features of toothpaste containing neem extract were found to be comparable to those of nonherbal toothpaste [123]. Neem is an herbal ingredient in toothbrushes and mouthwashes. Neem provides an effective solution for treating oral ulcers, preventing caries, and functioning as an oral deodorant and pain reliever for teeth [128].

### 7.12 Oak [*Quercus* spp. (Fagaceae)]

The medicinal uses of oak bark come from its various biological activities, such as reducing inflammation, fighting against microbes, neutralizing harmful substances, and destroying cells. These properties are attributed to the presence of compounds like tannins, flavonoids, phenolic acids, sterols, and triterpenoids in the bark. The oak species *Quercus petraea* L. and *Quercus robur* L. are the primary raw materials for obtaining oak bark. Tannins, which are abundant in oak bark, contribute to its astringent qualities. By forming long-lasting and insoluble compounds with proteins in microorganisms, tannins have bactericidal effects, interfering with bacterial growth and neutralizing toxins [38]. Additionally, oak bark affects capillaries by reducing permeability and controlling small-scale bleeding. Research suggests that an extract of oak bark, specifically *Q. infectoria*, may be used to combat dental caries and periodontitis because of its ability to kill bacteria like *S. mutans*, *P. gingivalis*, and *Streptococcus salivarius* [129].

### 7.13 Oregano [*O. vulgare* L.) (Lamiaceae)]

Oregano, an aromatic herb from the mint family, is one of the most widely traded culinary herbs. Numerous studies have investigated the potential health benefits of oregano, with particular emphasis on its phenolic compounds linked to antioxidant, anti-inflammatory, and antimicrobial properties [130]. Thymol (2-isopropyl-5-methylphenol) and carvacrol (5-isopropyl-2-methylphenol) are phenolic monoterpenes and represent the primary bioactive compounds in oregano [123]. Furthermore, oregano includes flavonoids such as apigenin, luteolin, and kaempferol [130]. Oregano extracts have shown potent antibacterial activity against multiple isolates of *Enterococcus*, *Streptococcus*, and *Lacticaseibacillus* [131,132]. A recent study by Yuan et al. [133] evaluated the *in vitro* antibacterial effectiveness of oregano essential oil against *Streptococcus mutans*. These findings suggest its potential as an antibacterial agent for preventing dental caries.

### 7.14 Rosemary [*Rosmarinus officinalis* L. syn. *Salvia rosmarinus* Spenn. (Lamiaceae)]

Rosemary has antioxidant, antibacterial, and antifungal properties, all of which are attributed to its phenolic compound constituents, mainly rosmarinic acid, caffeic acid, chlorogenic acid, and carnosic acid. Flavonoid-type polyphenols, including apigenin, luteolin, diosmin, and genkwanin (O-methylated flavone), are commonly present in rosemary [134]. It has demonstrated effectiveness against *E. coli*, *Scaphirhynchus albus*, *S. aureus*, and *Vibrio cholerae* [135]. Günther et al. [136] noted a strong antimicrobial impact of *R. officinalis* extract against both aerobic and anaerobic bacteria present in initial oral biofilms. The number of colony-forming units (CFUs) following the treatment of initial biofilms with *R. officinalis* extract was significantly lower than the number of CFUs in untreated biofilms. The dentifrice components included essential oils and extracts of rosemary for the formulation of mouthwashes and toothpastes. These formulations were found to be effective in preventing dental plaque, gingivitis, and bad breath [137].

### 7.15 Sage [*Salvia officinalis* L. (Lamiaceae)]

*S. officinalis* L. is a member of the tribe Mentheae in the mint family and is a popular herb commonly used in kitchens. *Salvia* belongs to the same botanical tribe as mint (*Mentha*), rosemary (*Rosmarinus*), thyme (*Thymus*), basil (*Ocimum*), oregano (*Origanum*), balm (*Monarda*), savory (*Satureja*), balm (*Melissa*), and catnip (*Nepeta*) [138]. Hence, it is not surprising that they share comparable ethnomedicinal features, including their closely related bioactive constituents. Since ancient times, sage has been recognized and utilized as both a culinary and medicinal herb. Its name stems from the Latin word salveo, meaning “to be healthy.” Sage phytoconstituents comprise polyphenols such as flavonoids and tannins, as well as terpenes/terpenoids. Beneficial compounds such as *α*- and *β*-thujones, 1,8-cineol, and camphor are found as primary components of the essential oil of sage [139].

Salvia extracts and infusions possess a range of beneficial properties, including anti-inflammatory, antibacterial, astringent, antimycosal, antihidrotic, and antilactative effects [140]. Sage infusions are commonly used to rinse the oral cavity and throat in cases of inflammation, including purulent diseases. In cases of oral disorders such as inflammatory diseases, sage leaves are frequently employed for their anti-inflammatory properties, although the effectiveness of these treatments may vary among randomized controlled trials [141,142,143].

### 7.16 Stevia [*Stevia rebaudiana* Bertoni (Asteraceae)]

Steviol glycosides are extracted from the leaves of stevia and used as substitutes for caloric sweeteners in beverages and foods. It exhibits antioxidant, anti-inflammatory, hypoglycemic, and antihypertensive properties [144]. The bioactive compounds present in the leaf extracts of stevia can be categorized into glycosides, alkaloids, polyphenols (i.e., flavonoids and tannins), saponins, quinones, and terpenoids. In addition, stevia leaf residue extract is a polyphenol-rich composition obtained from byproducts and includes caffeic acid, 3-*O*-caffeoylquinic acid, 3,4-*O*-dicaffeoylquinic acid, 3,5-*O*-dicaffeoylquinic acid, 4-*O*-caffeoylquinic acid, 4,5-*O*-dicaffeoylquinic acid, quercetin-3-*O*-rhamnoside, and quercetin [145].

A healthy substitute for sucrose is an important step to prevent not only adverse outcomes of impaired insulin function but also dental caries. Stevia extracts showed antibacterial activity – more relevant to our current focus on dentistry – against bacteria (*S. mutans*, *Streptococcus mitis*, *S. salivarius*, *Streptococcus rattus*, *Streptococcus cricetus*, *S. sobrinus*, *L. acidophilus*, *L. plantarum*, *Lacticaseibacillus casei*, and *Lactobacillus brevis*) involved in dental caries [146]. In another *in vitro* study, extracts of stevia leaf showed protective actions against causing agents of dental caries including *E. coli*, *S. mutans*, *Bacillus subtilis*, *S. aureus*, and *Curvularia lunata* [147]. Moreover, daily consumption of snacks containing stevia in comparison to sugar-based snacks for caries-related variables in 271 elementary school children (aged 6–9) resulted in reduced plaque acidogenicity and cariogenic microflora, thus reducing the risk of further development of caries [148]. There has been a growing interest in the antimicrobial and anti-plaque activity of stevia mouthrinse and chewing gums [149]. Mouthrinse containing 0.5% stevia extract improved salivary pH and buffering capacity in patients at high risk of caries [150].

### 7.17 Thyme (*Thymus* spp.)

Thymol and carvacrol, phenolic monoterpenes, are among the most important aromatic components of thyme. Carvacrol, an isomer of thymol, is a compound commonly found in the essential oils of the Mentheae tribe, including the two genera *Thymus* and *Mentha* [4,64,107,151]. The phytochemicals and antimicrobial properties of thyme (*Thymus* spp.) have recently been described by Soleimani et al. [4] and are not discussed further here. The antimicrobial and antioxidant potentials of thyme are strictly linked to phenolic compounds. The same reasons could explain the strong disinfecting and analgesic properties of thyme. In a recent study, it was reported that thyme-containing mouthwash is effective in reducing bad breath and gingivitis in gingivitis patients [152]. Thymol, a major bioactive component of thyme, precipitates in the form of blank crystals with a strong thyme smell. It can be used in 1–10% alcohol solutions to disinfect root canals for the treatment of pulp necrosis [67]. In addition to the above-mentioned applications, thyme essential oils are used for the production of dental and oral care products, such as hygiene products, as flavor-enhancing agents in toothpaste, chewing gums, and pharmaceuticals [4].

### 7.18 Turmeric [*Curcuma longa* L. (Zingiberaceae)]

The main component of the turmeric rhizome is a polyphenolic yellow pigment known as curcumin (diferuloylmethane). Curcumin is the most abundant phytoconstituent (75% of the extract), followed by dimethoxy-curcumin and bisdemethoxy-curcumin, all of which constitute 3–5% of the rhizome dry weight [153]. Turmeric is mainly used in food and has a variety of therapeutic applications for the treatment of various disorders, including dental and oral disorders. The potential therapeutic benefits of turmeric (curcumin) are broadly categorized as antioxidant, anti-inflammatory, antimicrobial, anticarcinogenic, antiarthritic, hypoglycemic, or thrombosuppressive [154]. In dentistry, the therapeutic benefits of curcumin-based formulations have been revealed for various oral conditions. Studies have shown that curcumin gel can be used for treating premalignant oral disorders (oral leukoplakia) [155] and minor recurrent aphthous stomatitis [156]. The antimicrobial effect of curcumin on root canals [157] and its anti-inflammatory and antiplaque effects on gingivitis management [158] have been demonstrated. This article suggests four areas for further research to advance the understanding of the therapeutic applications of curcumin for the treatment of dental and oral disorders: the management of chronic periodontitis [159], azole-resistant oral candidiasis [160], periodontal pockets [161], and oral cavity lesions [162].

### 7.19 Tulsi [*Ocimum tenuiflorum* L. or *Ocimum sanctum* L. (Lamiaceae)]

Tulsi, commonly referred to as “holy basil,” is a fragrant plant that possesses various therapeutic abilities. The bioactive compounds in the leaves and seeds of tulsi are categorized in order of importance as polyphenols (i.e., flavonoids and tannins), terpenes, glycosides, and steroids. Eugenol, methyl-eugenol, carvacrol, linalool, limatrol, apigenin, luteolin, quercetin, rosmrinic acid, ursolic acid, *α*- and *β*-pinenes, sterol, and caryophyllene are the main constituents of tulsi essential oil [163,164]. Each of these constituents displays a vast array of therapeutic benefits and contributes to the antimicrobial, anti-inflammatory, and antioxidant qualities of this plant. Pai et al. [164] documented the antibacterial effects of tulsi against *S. mutans*, *Streptococcus sanguis*, *S. mitis*, and *L. acidophilus*. In another study, the antibacterial activity of tulsi extracts was observed against *P. gingivalis*, Prevotella *intermedia*, and *A. actinomycetemcomitans*, with different inhibition zones [165].

The anti-inflammatory agents of *O. sanctum* aid in reducing gingival inflammation, a common feature of periodontal diseases. Tulsi extracts have been tested as mouthwash, toothpaste, and gel formulations. Due to its antimicrobial properties comparable to those of chlorhexidine, it may be recommended as a viable alternative for patients who are unable to utilize chlorhexidine [166]. A separate study assessed the effectiveness of mouthwashes containing tulsi and chlorhexidine, determining that both options equally reduce gingivitis and plaque, as measured by the plaque index, and reduce gingival bleeding and inflammation [167]. Therefore, tulsi has exceptional qualities that make it an effective long-lasting mouth freshener and oral disinfectant capable of eliminating bacteria responsible for dental caries, plaque, and calculus, ultimately safeguarding dental health.

### 7.20 Yarrow [*Achillea millefolium* L. (Asteraceae)]

Yarrow has traditionally been used to treat patients with ulcers and hemorrhages and to improve blood clotting. Preclinical studies have shown that it may have antiulcer, anti-inflammatory, antimicrobial, hepatoprotective, and anxiolytic properties [168,169]. The presence of polyphenols (including flavonoids and tannins) and terpene compounds in *A. millefolium* is responsible for its diverse pharmacological properties. The important components of yarrow include luteolin, apigenin, quercetin, chlorogenic acid, benzoic acid, cineole, spathulenol, borneol, pinene, germacrene, cis-carveol, camphor, azulen, achillin, and leucosis [168,170]. Hence, polyphenols, including flavonoids, phenol carbonic acids, and tannins, are the most pharmacologically important groups of bioactive compounds in yarrow. It is utilized as a mouthwash to aid in healing for mouth cuts resulting from surgery, toothaches, dental cleanings, and braces [171]. Persica, a trademark mouthwash from Iran, contains herbal ingredients such as miswak, mint, and yarrow.

### 7.21 Frankincense (olibanum) *Boswellia sacra* Flück (Burseraceae)

The *B. sacra* Flück tree, called Frankincense or olibanum, contains essential oil constituents that primarily include pentacyclic triterpenoid and various α- and β-boswellic acid derivatives. These derivatives include β-boswellic acid, acetyl-β-boswellic acid, 11-ketoβ-boswellic acid, acetyl-11-keto-beta-boswellic acid (AKBA), acetyl-α-boswellic acid, and α-boswellic acid [172]. AKBA biological properties include antioxidant, anti-aging, anti-inflammatory, anti-infective, neuroprotective, and anti-tumor effects [172]. Consistent with this notion, AKBA’s ability to regulate several signaling pathways, such as Nrf2/HO-1, extracellular signal-regulated kinase (ERK), and NFκB, has been demonstrated [173,174,175]. This may have implications in approaching neurodegenerative disease therapeutics, such as incensole acetate, the main component of Frankincense, along with AKBA. Chronically administered Frankincense compounds enhanced short-term memory in an AD rat model induced with streptozotocin. AKBA also proves to be significantly effective in conferring neuroprotection to substantia nigra dopaminergic neurons after neurointoxication with 6-OHDA, a rat model of PD [176]. Searching to understand the molecular mechanism of action of active components of Boswellia, a recent study from our laboratory investigated *B. sacra* Gum Resin effect on neuroinflammatory damage post-traumatic brain injury, where administration of 500 mg/kg resulted in significant protection against lipid peroxidation, antioxidant imbalance, cytokines release and autophagic flux alteration induced by TBI [177].

Both the essential oils and the isolated boswellic acid of *Boswellia* exhibit antimicrobial properties. Out of the four major *β*-boswellic acids, AKBA has reliably shown the strongest antibacterial effects [178]. Therefore, it makes sense to include frankincense and its derivative boswellic acids in mouthwashes and toothpaste. Frankincense oil, known for its antimicrobial properties, is used as a mouthwash solution because the essential oil, derived from the resin of the *Boswellia* tree, is effective in inhibiting pathogenic oral bacteria, specifically, *P. gingivalis*, *S. mutans*, and *Fusobacterium nucleatum* [172].

### 7.22 Medical mushrooms

Mushrooms have been used in traditional medicine for thousands of years, owing to their content of phenolic compounds (caffeic acid, gallic acid, cinnamic acid, melatonin, *p*-hydroxybenzoic acid, *p*-coumaric acid, protocatechuic acid) and other antioxidant molecules, such as ergothioneine and glutathione [179]. Mushrooms are not only rich sources of enzymatic and non-enzymatic antioxidants but also contain fiber, essential amino acids, and polysaccharides. Several mushroom species, such as *Hericium erinaceus*, *Coriolus versicolor* (syn. *Trametes versicolor*, *Polyporus versicolor*), *Lentinula edodes*, *Ganoderma lucidum*, *Agaricus bisporus*, *Grifola frondosa*, *Polyporus umbellatus*, *Phellinus linteus*, and *Pleurotus ostreatus*, are known for their medicinal properties and a variety of applications. Medicinal mushrooms stand as a rich source of phytochemical constituents with potent benefits for oral and general health.

*H. erinaceus* (Bull.) Pers. (*HE*) contains erinacines, which cross the blood-brain barrier, and hence may exert neuroprotective functions [180]. In mouse models of AD, *HE* induces decreased deposition of Aβ and reduces the levels of reactive astrocytes and microglia [181]. *HE* increased the production of nerve growth factor (NGF) which inhibited the enzymes choline acetyltransferase and acetylcholinesterase responsible for cholinergic neuron dysfunction in AD [182]. *HE* also suppressed apoptosis in pheochromocytoma 12 cells by inhibiting oxidative stress induced by the neurotoxin di(2-ethylhexyl) phthalate (DEHP) and stabilizing the mitochondrial membrane potential. This cytoprotective effect is thought to be due to its ability to reduce intracellular levels of ROS, maintain respiratory complex activity, and stabilize mitochondrial membrane potential [183]. Furthermore, *HE* modulates the BDNF/TrkB/PI3K/Akt/GSK-3β pathway and induces antidepressant-like effects in a mouse model of anxiety and sleep disturbance. Amycenone^®^ and *HE* extract derived from the fruiting body, showed antidepressant and anxiolytic effects in an open field test, tail suspension test, and forced swimming in an animal model of lipopolysaccharide-induced inflammation-related depression [184].

In addition to diterpenoids like erinacines, *HE* contains other major bioactive compounds such as erinacerins, erinaceolactones, hericerins, sterols, glycoproteins, and polysaccharides [185]. Consequently, the key phytochemicals in *HE* are categorized into terpenoids, polyphenols, cerebrosides, and sterols. A food-grade laccase from *HE* incorporated into mouthwash effectively inhibited *S. mutans*, *S. aureus*, and *C. albicans*, with lower cytotoxicity than chlorhexidine mouthwash on human gingival fibroblasts. The antifungal effects of *HE* also have also been demonstrated. For example, 2-chloro-1,3-dimethoxy-5-methyl benzene, a derivative isolated from the mycelia of *Hericium* spp. is effective against *C. albicans* and *Cryptococcus neoformans* [186]. The laccase mouthwash also demonstrated significant tooth-whitening effects compared to the control, suggesting it could be a safe, natural alternative to chemical mouthwashes [187]. As with *HE*, *C. versicolor* (L.) Quél. (*CV*) is also a well-known medicinal mushroom that has been shown to increase levels of the antioxidant enzymes superoxide dismutase and catalase along with the reduction of inflammatory cytokines such as TNF-α and IL-1β; the modulation of these parameters was associated with the improvement in spatial memory in AD model mouse [188]. In addition, repeated administration of *CV* biomass preparations was found to upregulate lipoxin A4 (LXA4) in cortical and hippocampal regions of the rat brain, resulting in increased levels of Hsp72, heme oxygenase-1, thioredoxin, and others [189]. LXA4 is a potential therapeutic target for neurodegenerative damage in the hypothesis that neurodegenerative diseases are caused by oxidative stress-induced neuroinflammation. In patients with Meniere’s disease, a disease characterized by oxidative stress, *CV* biomass showed a reduction of oxidative stress markers (protein carbonyls, hydroxynonenals, and F2-isoprostanes) in peripheral blood and an up-regulation of lymphocyte vitagine (HO-1, Hsp70, Trx, sirtuin-1, γ-glutamate–cysteine lyase), indicating a response against the pro-oxidant state in cells [190]. In addition, *CV* biomass caused increased β-catenin levels in the nucleus and cytoplasm in the mouse hippocampus and a significant increase in the volume, length, and branching of dendrites of immature hippocampal neurons, suggesting that it may stimulate neurogenesis [191]. The results illustrated above demonstrate *HE* and *CV* are endowed with multiple neuroprotective properties, which might counteract some molecular mechanisms underlying AD pathophysiology. A recent study suggested the role of *HE* mycelium in promoting oligodendrocytes (OLs) differentiation and production of myelin basic protein *in vitro* and *in vivo*. Erinacin A and erinacin S, which are found in *HE* mycelia and cross the brain barrier, induce maturation and myelination of dilated glial cells, and promote action potential propagation [192]. Since OL maturation, myelination, and brain connectivity were reported to be impaired in AD [193], future studies should evaluate the potential of *HE* to regulate such pathological mechanisms in AD. In addition, *HE* mycelia rich in erinacin A have been reported to enhance NGF activity and promote neurite outgrowth [194]. This is an important process in neurogenesis and is known to be impaired by environmental and genetic risk factors associated with AD. As mentioned above, *CV* improved the Wnt/β-catenin pathway and dendrite formation which is known to impair AD [195,196]. Thus, supplementation with *HE* and *CV* may induce beneficial synergistic effects on brain development and neurodevelopment. Finally, these mushrooms may promote resilience during brain development by increasing redox potential through neurohormesis mechanisms such as the upward regulation of vitagenes, represented by Nrf2 [194,197]. *CV* is a strong candidate for oral health care drugs and products because it is a rich source of bioactive ingredients. The *CV* extracts showed antimicrobial activities against *S. aureus*, *Streptococcus mutans*, *S. sanguinis*, *S. sobrinus*, *Streptococcus ratti*, *S. criceti*, *A. actinomycetemcomitans*, *Actinomyces israeli*, and *Actinomyces viscosus*. Therefore, *CV* could be used as a natural oral antimicrobial source against oral pathogens [198].

*Lentinus edodes* (Berk.), commonly known as Shiitake, is an edible mushroom renowned for its antimicrobial, anti-inflammatory, and anti-plaque properties [199]. *L. edodes* (shiitake mushroom) is the second most important fungus, following *A. bisporus* (button mushroom), in global production. From the oral health point of view, *L. edodes* extracts showed anti-caries and anti-gingivitis effects, as well as the decline of oral biofilm formation [200]. Comparing Shiitake mushroom mouthwash with chlorhexidine, the gold standard for treating moderate to severe gingivitis, reveals that while chlorhexidine remains superior, Shiitake mushroom mouthwash also demonstrates promising anti-inflammatory and anti-plaque effects [199]. A low molecular mass (LMM) fraction from an aqueous shiitake mushroom extract (*L. edodes*) has demonstrated antiadhesive, antimicrobial, and antiplaque activities *in vitro* against various oral pathogens. This LMM fraction was incorporated into a mouthrinse and tested on 30 young volunteers, yielding promising results compared to two control groups: placebo and Listerine [201].

*G. lucidum* (Curtis) P. Karst, called Reishi mushroom, is one of the most famous ancient medicinal mushrooms. *G. lucidum* contains a range of bioactive compounds, including polyphenols, triterpenoids, and polysaccharides. It is known for its potential health benefits, including the anti-tumor effects by inducing apoptosis, enhancement of immune cell activity, interfering with key cancer cell signaling pathways, and inhibiting angiogenesis [202]. The antimicrobial properties of *G. lucidum* have been demonstrated against bacteria and fungi relevant to oral health [203].

*A. bisporus* JE Lange, called button mushroom, is the most widely planted and consumed mushroom globally. Additionally, this mushroom has been related to various health benefits, such as anti-inflammatory and antioxidant attributes, as well as potential support for the immune system. The ethanol extracts of both *A. bisporus* and *L. edodes* have demonstrated comparable antibacterial activity against *S. aureus*, *Acinetobacter baumannii*, *E. faecalis*, and *Klebsiella pneumoniae* [204].

*G. frondosa* (Dicks.) Gray, commonly known as the maitake mushroom, is an edible, nutritional, and medicinal fungus. The complex chemical composition of *G. frondosa* is attributed to its diverse bioactive compounds, including polysaccharides, ergosterol, β-D-glucans, dextrin, oligofructose, lactulose, various phenolic compounds, and triterpenes, all of which contribute to its health benefits [205]. The various polysaccharide fractions extracted from *G. frondosa* exhibit diverse bioactive properties. β-Glucans isolated from maitake demonstrate anti-inflammatory activity induced by lipopolysaccharide in the macrophage cell line RAW264.7, mediated through interaction with toll-like receptor 2 rather than with dectin-1 or complement receptor 3 [206].

The species *Polyporus*, *Phellinus*, and *Pleurotus* are significant sources of therapeutic substances, demonstrating important anti-inflammatory, antioxidant, and antitumor effects. Overall, various metabolites from these mushrooms, such as polysaccharides, lipopolysaccharides, glycoproteins, terpenoids, and phenolic compounds, have been identified as effective agents for enhancing the immune system.

To summarize and answer the inquiry what are the oral health benefits of the major and most important phytochemicals? Polyphenols and terpenes offer diverse oral health benefits, including antimicrobial action to combat pathogens like *S. mutans*, antioxidant protection against oxidative stress, and anti-inflammatory effects to reduce gum disease. They also support wound healing, regulate cell signaling pathways and exhibit anticancer and anticariogenic attributes. Together, these compounds help prevent oral diseases, promote tissue repair, and maintain a balanced oral microbiome. It is noteworthy that, beyond their health-promoting effects, certain terpenoids serve as precursors to essential vitamins, such as β-carotene (vitamin A), tocopherols (vitamin E), and ubiquinone (coenzyme Q) [207]. Therefore, the application of polyphenols and terpenes compounds in everyday human life and oral health is widely used in pharmaceutical, nutraceutical, food, and beverage products.

## 8 Conclusions and future perspectives

There is a growing interest in the utilization of herbal products in alternative medicine as a means to prevent and treat oral and dental issues. However, recent attention has shifted toward the question of whether medicinal plants, including fresh parts, dried parts, extracts, and essential oils, might play a pivotal role in promoting optimal oral health. This review has endeavored to bridge this knowledge gap by consolidating evidence on the potential benefits of herbal polyphenols, terpenes, and terpenoids in maintaining oral health. In addition, this review offers a comprehensive overview of the current state of knowledge in the field, encompassing the pharmacological properties of these key phytochemicals, their mode of action, and their efficacy in the prevention and treatment of oral and dental diseases and conditions. Among the herbs discussed, miswak, neem, clove, peppermint, and tulsi have emerged as particularly promising options for promoting oral health. They exhibit mouth-freshening properties, act as natural disinfectants, and display antibacterial effects that can aid in the prevention of dental caries, plaque buildup, and calculus. In summary, this review underscores the potential of herbal polyphenols, terpenes, and terpenoids as valuable allies for improving oral health. This study contributes to the growing body of knowledge in the field, offering insights into the therapeutic potential of these natural compounds in maintaining healthy teeth and oral cavities.

Further research is imperative to validate these promising findings, especially through ongoing human trials. The potential health advantages of herbs and spices have not received extensive scrutiny, leading to a lack of consistency and comparability among existing research. This variability poses challenges in developing standardized clinical protocols. To address issues of safety, appropriate dosage, bioavailability, and efficacy of herbs and spices, it is essential to conduct clinical studies employing placebo controls. One unresolved question in this field pertains to the feasibility of formulating a blend of various herbs into an effective dental healthcare remedy. In the current age of increasing antibiotic resistance, there is substantial potential for in-depth investigations into the antimicrobial properties and activities of herbal plants.

## Abbreviations

ATP: adenosine triphosphate
CTs: condensed tannins
E4P: d-erythrose-4-phosphate
DAHP: 3-deoxy-d-arabino-heptulosonic acid 7-phosphate
PALs: phenylalanine ammonia-lyases
PEP: phosphoenolpyruvic acid
*ρ*HCA: *ρ*-hydroxycinnamic acid
PACs: proanthocyanidins
RNS: reactive nitrogen species
ROS: reactive oxygen species
TALs: tyrosine ammonia-lyases
